# Distinct Clinical and Outcome Profiles Across Six Subtypes of Acute Gastrointestinal Bleeding: A Comprehensive Analysis of 1021 Patients

**DOI:** 10.3390/jcm15051998

**Published:** 2026-03-05

**Authors:** Nóra Vörhendi, Levente Frim, Orsolya Anna Simon, Eszter Boros, Brigitta Teutsch, Dániel Pálinkás, Edina Tari, Dávid Berki, Patrícia Kalló, Edina Ecsedy, Endre Botond Gagyi, Armand Csontos, Zoltán Sipos, Nelli Farkas, Áron Vincze, Ferenc Izbéki, Andrea Szentesi, Roland Hágendorn, Imre Szabó, Péter Hegyi, Bálint Erőss

**Affiliations:** 1 Institute for Translational Medicine, Medical School, University of Pécs, 7624 Pecs, Hungary; levi.frim@gmail.com (L.F.); orsi.simon55@gmail.com (O.A.S.); boroseszter987@gmail.com (E.B.); teutschbrigitta@gmail.com (B.T.); berkidavid93@gmail.com (D.B.); kallo.patricia97@gmail.com (P.K.); edina_ecsedy@yahoo.com (E.E.); csontos.armand@gmail.com (A.C.); zolike.sipos@gmail.com (Z.S.); nelli.farkas@aok.pte.hu (N.F.); szentesiai@gmail.com (A.S.); hegyi2009@gmail.com (P.H.); eross.balint@pte.hu (B.E.); 2 Clinical Center, Division of Gastroenterology, First Department of Medicine, Medical School, University of Pécs, 7624 Pecs, Hungary; vincze.aron@pte.hu (Á.V.); hagendornroland@gmail.com (R.H.); szaboimi@yahoo.com (I.S.); 3 First Department of Internal Medicine, Fejér County Szent György University Teaching Hospital, 8000 Szekesfehervar, Hungary; fizbeki@gmail.com; 4 Centre for Translational Medicine, Semmelweis University, 1085 Budapest, Hungary; edina.tari@gmail.com (E.T.); endre.gg@gmail.com (E.B.G.); 5 Department of Gastroenterology, Central Hospital of Northern Pest, Military Hospital, 1062 Budapest, Hungary; danipalinkas@gmail.com; 6 Institute of Bioanalysis, Medical School, University of Pécs, 7624 Pecs, Hungary; 7 Institute of Pancreatic Diseases, Semmelweis University, 1083 Budapest, Hungary

**Keywords:** gastrointestinal hemorrhage, upper gastrointestinal bleeding, lower gastrointestinal bleeding, variceal bleeding, small bowel bleeding, iatrogenic disease, endoscopic hemostasis, hospital mortality

## Abstract

**Background**: Acute gastrointestinal bleeding (GIB) remains a major clinical emergency with substantial morbidity, mortality, and healthcare burden. We aimed to provide a comprehensive characterization of all GIB subtypes, including iatrogenic bleeding, which is underrepresented in previous studies. **Methods**: In this ambidirectional cohort study, 1021 consecutive adults with overt GIB were enrolled from two Hungarian tertiary centers. Standardized data collection included demographics, comorbidities, medication use, bleeding source, and in-hospital outcomes: mortality, rebleeding, intensive care unit (ICU) admission, length of hospitalization (LoH), endoscopic evaluation and haemostatic intervention, red blood cell transfusion, and surgical intervention. Group comparisons were performed using appropriate statistical tests, and survival was analysed using Kaplan–Meier curves (R v4.4.2; *p* < 0.05). **Results**: Non-variceal upper GIB was the most common subtype (51.0%), followed by lower GIB (29.7%), variceal GIB (8.9%), small bowel bleeding (2.3%), and iatrogenic bleeding (7.5%). Overall, in-hospital mortality was 10.6%, highest in variceal bleeding (22%). Rebleeding occurred in 5.3% of cases, most frequently in variceal bleeding. ICU admission was required in 8.9% of patients, again, most common in variceal bleeding (21.6%). The median LoH was 7 days (IQR 4–10), significantly shorter in cases of intraprocedural iatrogenic bleeding. Endoscopy was performed in 91% of cases, with haemostatic intervention in 57%. Surgery was required in 3.4% of patients. **Conclusions**: Gastrointestinal bleeding represents a heterogeneous clinical entity with distinct outcome profiles across subtypes. Variceal bleeding was associated with the most unfavorable outcomes, whereas intraprocedural iatrogenic bleeding had a favorable course. These findings support subtype-specific clinical management and warrant validation in larger multicenter cohorts.

## 1. Introduction

Acute gastrointestinal bleeding (GIB) remains a frequent and clinically significant medical emergency, associated with considerable morbidity, mortality, and healthcare resource utilization worldwide [1]. Prompt identification, accurate classification, and targeted management are crucial for improving patient outcomes. GIB can be classified into distinct subtypes: non-variceal upper gastrointestinal bleeding (NVUGIB), variceal upper gastrointestinal bleeding (VUGIB), small bowel bleeding (SBB), and lower gastrointestinal bleeding (LGIB).

NVUGIB is the most prevalent subtype of acute gastrointestinal bleeding, constituting approximately 60–70% of all GIB cases. The annual incidence is reported between 80 and 150 per 100,000 persons. Common etiologies include peptic ulcer disease, erosive gastritis, and gastrointestinal malignancies [2]. VUGIB accounts for approximately 10–30% of gastrointestinal bleeding episodes, predominantly associated with portal hypertension secondary to liver cirrhosis. Its incidence is estimated at around 10–20 per 100,000 population annually, significantly contributing to morbidity and mortality in affected patients [3]. SBB accounts for approximately 5–10% of GIB cases, with an annual prevalence of 2–8 per 100,000 persons. Due to its anatomical location between the ligament of Treitz and the ileocecal valve, diagnosis and treatment of SBB often require specialized imaging modalities and advanced intervention techniques [4]. LGIB, defined as bleeding arising distal to the ileocecal valve (colon and rectum), accounts for 20–30% of acute gastrointestinal bleeding cases. Its incidence is approximately 20–30 per 100,000 individuals annually, with leading causes including diverticular disease, angiodysplasia, and colorectal malignancies [5].

Iatrogenic gastrointestinal bleeding, resulting from diagnostic or therapeutic interventions such as endoscopic biopsy, polypectomy, endoscopic mucosal resection, or anticoagulation therapy, remains comparatively under-characterized in the literature. Although post-endoscopic bleeding is a recognized complication, most studies have examined it in specific procedural cohorts rather than within the broader clinical context of GIB. As a result, available data on incidence, therapeutic approaches, and clinical outcomes are heterogeneous and frequently incomplete [6,7]. This lack of standardized outcome characterization limits the ability to evaluate iatrogenic bleeding as a distinct clinical entity.

Endoscopy remains the primary diagnostic and therapeutic modality in the management of GIB, although some cases require angiographic or surgical intervention. In addition, hospital resource utilization, particularly length of hospitalization (LoH) and intensive care unit (ICU) admission, significantly impacts patient outcomes and contributes to healthcare burden.

Several extensive national audits have provided robust real-world data on gastrointestinal bleeding; however, these cohorts were restricted to specific anatomical subsets and applied non-uniform outcome measures. The UK National Audit of Acute Upper Gastrointestinal Bleeding focused exclusively on upper GI presentations and primarily assessed mortality and service organization [8]. In contrast, the nationwide audit of lower gastrointestinal bleeding emphasized transfusion requirements, rebleeding, and procedural timing [9]. Due to these differences in outcome focus, neither study allows for consistent comparison across the full spectrum of gastrointestinal bleeding.

A unified evaluation of all major gastrointestinal bleeding subtypes within a single standardized framework is therefore necessary to better understand clinical and outcome differences between bleeding entities and to support subtype-oriented clinical decision-making. The present study aims to address this gap by comprehensively analysing consecutive acute GIB presentations at two Hungarian tertiary care centres, emphasizing clinical characteristics and patient outcomes across all major bleeding subtypes. By applying uniform outcome definitions and consistently evaluating key clinical endpoints across six clinically relevant bleeding categories, we provide a comprehensive descriptive comparative framework within a real-world population that has not been available in previous national audits. Furthermore, by benchmarking our findings against international data, our results may serve as a reference for understanding patterns of morbidity, mortality, and healthcare resource utilization in clinical practice.

## 2. Materials and Methods

### 2.1. Study Design and Setting

This study was designed as an ambidirectional cohort analysis, incorporating both retrospectively identified and prospectively collected cases from the Hungarian Gastrointestinal Bleeding Registry. Between 6 October 2019 and 7 April 2022, the registry accumulated data from two tertiary centres, the First Department of Internal Medicine, University of Pécs, and the First Department of Internal Medicine, St. George University Teaching Hospital of County Fejér, Székesfehérvár. The registry received ethical permission from the Scientific and Research Ethics Committee of the Medical Research Council (24433-5/2019/EÜIG) in 2019. All participants provided written informed consent before being enrolled in the registry. The manuscript was prepared according to the Strengthening the Reporting of Observational Studies in Epidemiology (STROBE) 2007 checklist [10] (Supplementary Table S1).

### 2.2. Participants

All consecutive adult (≥18-year-old) patients with signs of overt GIB (melena, haematochezia, hematemesis, clinically significant intraprocedural bleeding during endoscopy) from both centres were enrolled into the registry. All patients involved in the registry are included in this analysis. Following the onset of overt gastrointestinal bleeding, most patients were admitted through the emergency department to the Gastroenterology Unit, Internal Medicine Ward, or High Dependency Unit. Patients who developed intraprocedural bleeding during endoscopy or experienced bleeding while hospitalized for non-gastroenterological conditions were also included in the registry.

### 2.3. Data Sources and Data Collection

Patient data were collected within the Hungarian Gastrointestinal Bleeding Registry coordinated by the Translational Medicine Centre, which provides standardized case report forms (CRFs), predefined variable definitions, and harmonized documentation methods. The registry protocol and data collection forms are publicly accessible at https://tm-centre.org/hu/kutatas/regiszterek/gib-regiszter (accessed on 1 March 2026). Data were organized in a secure, password-protected online database (Electronic Clinical Data Management System, ECDMS).

A structured four-level validation process was implemented to ensure data reliability and consistency. Clinical variables were initially recorded at the bedside using standardized paper CRFs by trained research personnel, based on patient interviews, information from relatives when necessary, consultation with the treating physician, and review of the electronic medical record. Data were subsequently entered into the ECDMS and independently verified against source documentation by a physician or PhD investigator. A central registry coordinator performed cross-center completeness and consistency checks, followed by final clinical validation by a senior gastroenterologist. Discrepancies were resolved through re-evaluation of source data and consensus discussion among investigators. This multi-stage verification process resulted in complete data availability for all analyzed variables.

The following data categories were recorded:

- Demographics: age, sex, and the location where bleeding was first detected (community-onset vs. in-hospital).
- Risk factors: current and past smoking; current and past alcohol consumption.
- Medication use: all medications taken at home before admission, including antiplatelet agents, anticoagulants, NSAIDs, proton pump inhibitors (PPIs) or H2-receptor antagonists, and corticosteroids.
- Hemodynamic status on admission: presence or absence of hemodynamic instability
- Comorbidities: diabetes mellitus (type 1 or type 2), hypertension, liver disease and cirrhosis, history of malignant tumors, pulmonary diseases, ischemic heart disease, acute myocardial infarction, atrial fibrillation or flutter, transient ischemic attack, stroke, pulmonary embolism, deep vein thrombosis, chronic renal failure, peptic ulcer disease, and previous manifest gastrointestinal bleeding.
- Clinical outcomes: in-hospital mortality, in-hospital rebleeding, need for intensive care unit (ICU) admission, length of hospitalization (LoH), performance of endoscopy, use of endoscopic haemostatic interventions (including method of intervention), red blood cell (RBC) transfusion during hospitalization, identification of malignant lesions as the source of bleeding, and need for surgical intervention.

The definitions used in this study are summarized in Supplementary Table S2.

### 2.4. Data Quality

A standardized four-stage data validation protocol was implemented to ensure the reliability of the data. Each variable underwent multiple verification rounds before inclusion in the final dataset, resulting in an overall data completeness rate of 100%. Additional details are provided in Supplementary Table S3.

### 2.5. Patient Subgroups

Based on the source of bleeding, participants were placed into one of six groups for analysis, namely the non-variceal upper GIB (NVUGIB), variceal upper GIB (VUGIB), small bowel bleeding (SBB), lower GIB (LGIB), intraprocedural iatrogenic GIB (IIGIB), and delayed iatrogenic GIB (IDGIB) groups.

The NVUGIB group included patients with a bleeding source proximal to the ligament of Treitz, excluding variceal bleeding. The VUGIB group comprised patients with oesophageal and gastric variceal bleeds. The SBB group included jejunal and ileal bleeding confirmed by capsule endoscopy or CT/MR angiography. The LGIB group contained patients with a colonic or rectal bleeding source. The IIGIB group consisted of patients whose clinically significant bleeding was detected during the endoscopic procedure and required hospital admission, while the IDGIB group included patients who developed overt GIB following the completion of the procedure.

When patients had multiple sources of bleeding, the lead investigator determined the primary cause by reviewing the patient’s history, endoscopic findings, clinical presentation, and potential pathological opinion.

### 2.6. Statistical Methods

Descriptive statistics were applied to summarize the data. Continuous variables are presented as the mean ± standard deviation (SD) or median and interquartile range (IQR), as appropriate, while categorical variables are reported as counts and percentages. Group comparisons for continuous variables were performed using one-way ANOVA; where significant, pairwise post hoc comparisons were conducted using Tukey’s test. Categorical variables were compared using Pearson’s chi-squared test or Fisher’s exact test when expected cell counts were low; pairwise comparisons were adjusted using the Benjamini–Hochberg procedure to control the false discovery rate. Survival analysis for in-hospital mortality was conducted using Kaplan–Meier estimates, with the length of hospital stay serving as the time variable. Differences between groups were assessed using the log-rank test. A two-tailed *p*-value < 0.05 was considered statistically significant. All analyses were performed using R (version 4.4.2; R Foundation for Statistical Computing, Vienna, Austria).

## 3. Results

Between 6 October 2019 and 7 April 2022, a total of 1021 consecutive GIB cases were entered into the registry and included in the present analysis.

### 3.1. Basic Characteristics

Patient characteristics, including demographic data, risk factors, haemodynamic status on admission, previous medication, and comorbidities, are summarized in Table 1.

Concerning the origin or etiology of GIB in this population, our results revealed that the most common cause of acute GIB was NVUGIB (51.03%), while VUGIB, SBB, and LGIB were the cause in 8.9%, 2.25%, and 29.68%, respectively. During this period, among the 1021 studied cases, there were 77 iatrogenic bleeders (2.74% IIGIB and 4.8% IDGIB).

### 3.2. Outcomes

The outcomes are summarized in Table 2.

#### 3.2.1. In-Hospital Mortality Rates and Causes of Death

**Figure 1:**
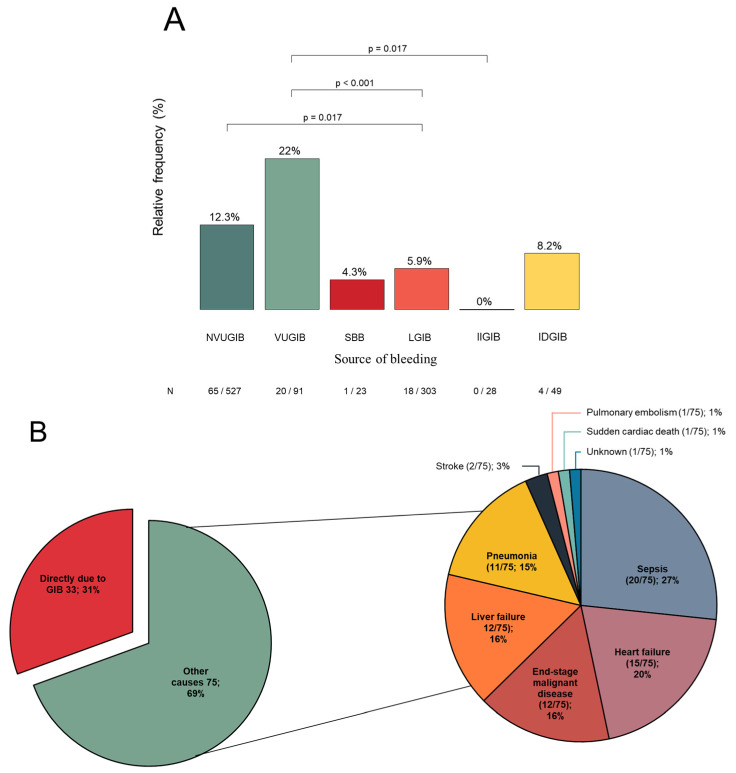
(**A**): In-hospital mortality rates in the different sources of GIB and the significant differences between the bleeding groups GIB: acute gastrointestinal bleeding, NVUGIB: non-variceal upper gastrointestinal bleeding, VUGIB: variceal upper gastrointestinal bleeding, SBB: small bowel bleeding, LGIB: lower gastrointestinal bleeding, IIGIB: iatrogenic intraprocedural gastrointestinal bleeding, IDGIB: iatrogenic delayed gastrointestinal bleeding. (**B**): The causes of death in acute gastrointestinal bleeding.

The overall in-hospital mortality rate in the study population was 10.6% (108/1021). Mortality was highest in the VUGIB group (22.0%, 20/91), which was significantly higher compared with the NVUGIB and LGIB groups. The in-hospital mortality rates in the NVUGIB, SBB, and LGIB groups were 12.3% (65/527), 4.3% (1/23), and 5.9% (18/303), respectively. No deaths occurred in the IIGIB group, whereas the mortality rate in the IDGIB group was 8.2% (4/49). Significant differences in mortality were observed between the NVUGIB and LGIB groups, between the VUGIB and IIGIB groups, and between the VUGIB and LGIB groups (Figure 1A).

Causes of death were reviewed by a gastroenterologist and a pathologist and categorized into deaths directly attributable to GIB and deaths due to other causes. GIB was the direct cause of death in 31% (33/108) of cases. The remaining 69% (75/108) of fatalities resulted from complications such as sepsis, progression of underlying chronic disease (e.g., liver or heart failure), end-stage malignant disease, pneumonia, or thromboembolic events (Figure 1B).

**Figure 2:**
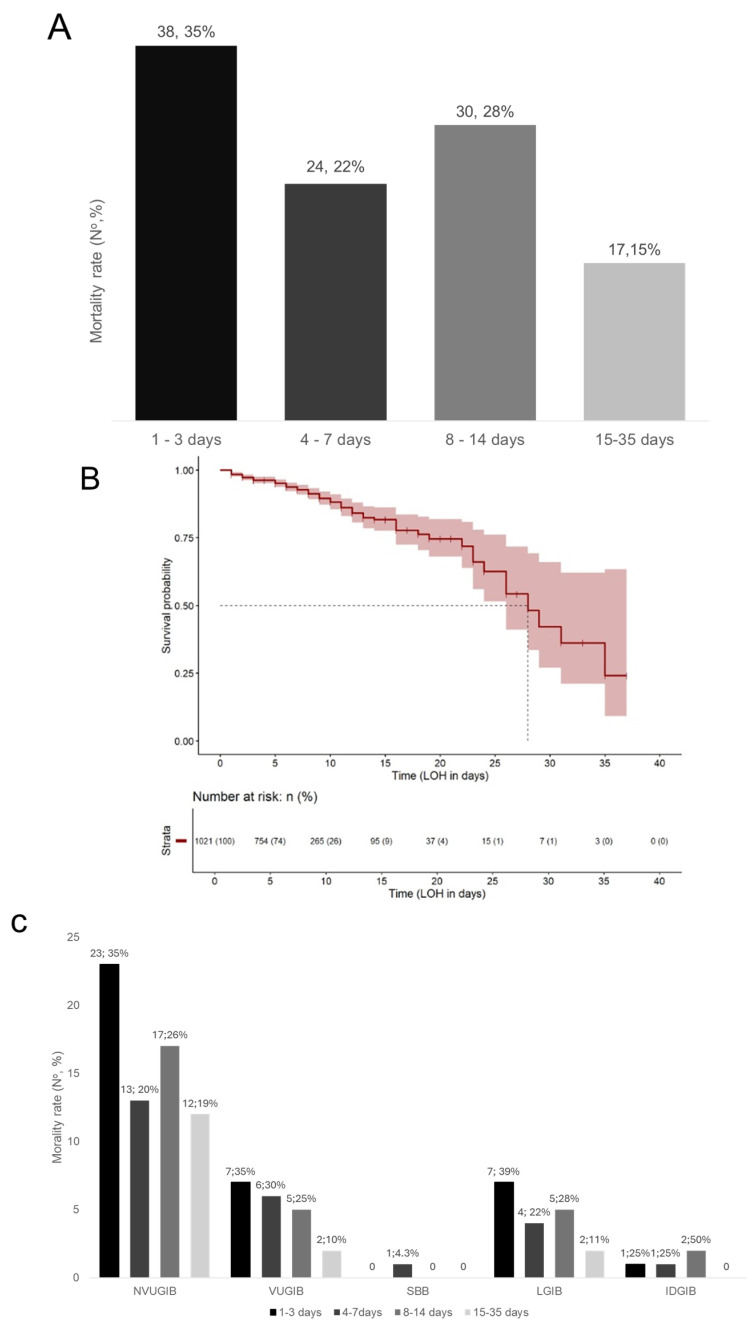

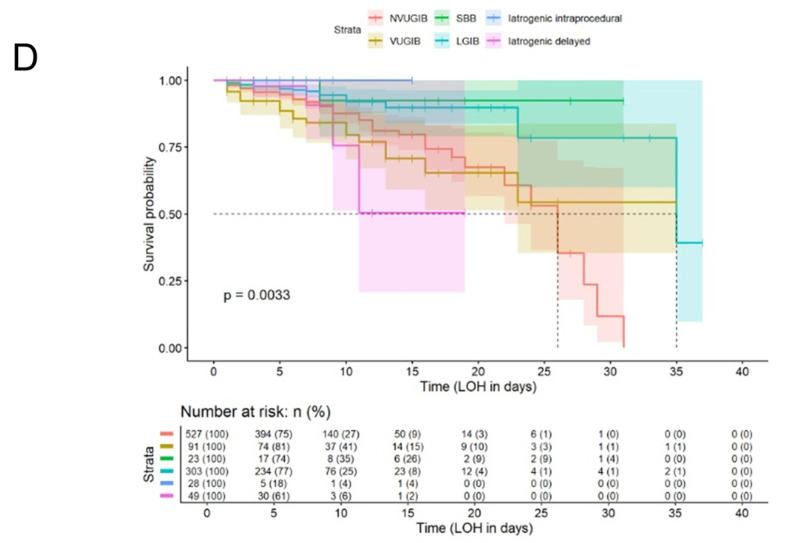
(**A**): Shows the number and rate of deaths during hospitalization in the all-cause acute gastrointestinal bleeding cohort; (**B**): The Kaplan–Meier curve illustrates the overall survival probability. LoH: length of hospitalization; (**C**): Shows the number and rate of deaths during hospitalization stratified by the source of acute gastrointestinal bleeding. NVUGIB: non-variceal upper gastrointestinal bleeding, VUGIB: variceal upper gastrointestinal bleeding, SBB: small bowel bleeding, LGIB: lower gastrointestinal bleeding, IDGIB: iatrogenic delayed gastrointestinal bleeding. (**D**): Kaplan–Meier curves depict survival probability across the subgroups. NVUGIB: non-variceal upper gastrointestinal bleeding, VUGIB: variceal upper gastrointestinal bleeding, SBB: small bowel bleeding, LGIB: lower gastrointestinal bleeding, IDGIB: iatrogenic delayed gastrointestinal bleeding, IIGIB: iatrogenic intraprocedural gastrointestinal bleeding, LoH: length of hospitalization.

Analysis of the temporal distribution of mortality demonstrated that deaths occurred predominantly early after admission. 35% (38/108) of all deaths occurred within the first 3 days, and 57% (62/108) occurred within the first 7 days. An additional 28% (30/108) of deaths occurred between days 8–14, while the remaining 15% (17/108) occurred after day 14. This pattern indicates a front-loaded mortality curve, in which the highest risk of death is concentrated during the initial phase of hospitalization (Figure 2A,B). A similar early-phase clustering was observed across all bleeding subtypes (Figure 2C,D), confirming that this temporal mortality pattern was consistent regardless of the bleeding source.

#### 3.2.2. In-Hospital Rebleeding

**Figure 3:**
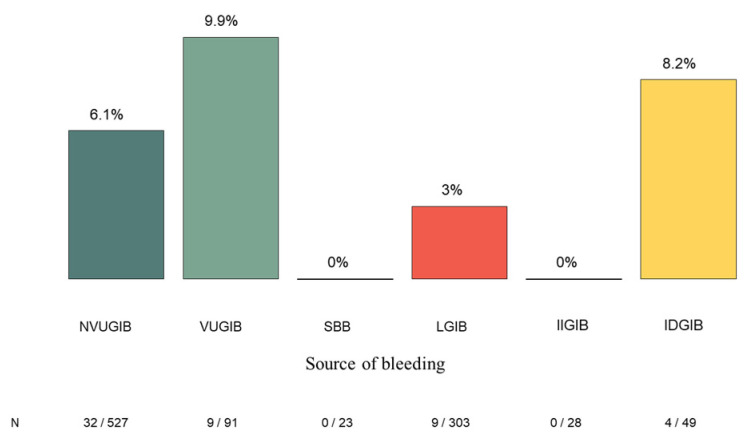
The in-hospital rebleeding rate and the significant differences between the bleeding groups. NVUGIB: nonvariceal upper gastrointestinal bleeding, VUGIB: variceal upper gastrointestinal bleeding, SBB: small bowel bleeding, LGIB: lower gastrointestinal bleeding, IIGIB: iatrogenic intraprocedural gastrointestinal bleeding, IDGIB: iatrogenic delayed gastrointestinal bleeding.

Rebleeding during hospitalization occurred in 54 of 1021 patients (5.3%). The highest rebleeding rate was observed in the VUGIB group (9.9%, 9/91), followed by the IDGIB group (8.2%, 4/49) and the NVUGIB group (6.1%, 32/527). Rebleeding was less frequent in LGIB (3.0%, 9/303), and no rebleeding events were recorded in the SBB (0/23) or IIGIB (0/28) groups. Although the distribution of rebleeding rates varied across subgroups, no statistically significant differences were observed between the groups (Figure 3).

#### 3.2.3. Need for Intensive Care

**Figure 4:**
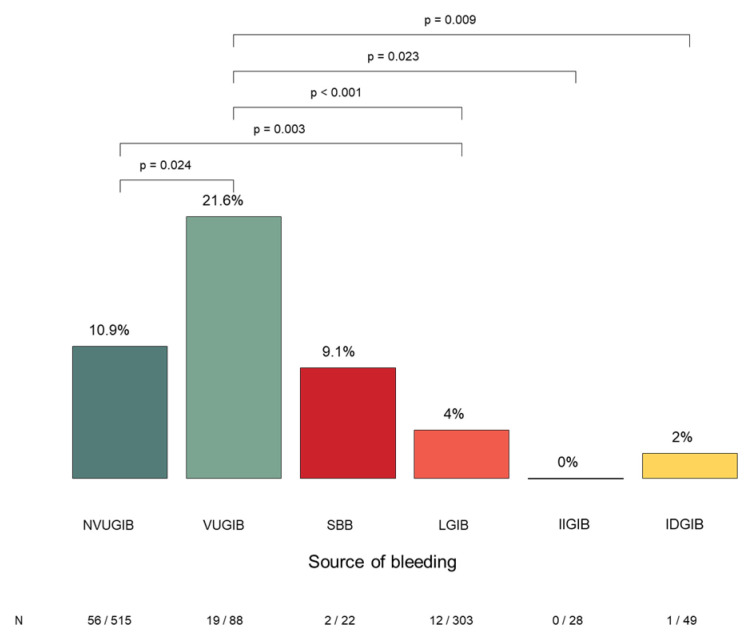
The need for intensive care and the significant differences between the bleeding groups. NVUGIB: nonvariceal upper gastrointestinal bleeding, VUGIB: variceal upper gastrointestinal bleeding, SBB: small bowel bleeding, LGIB: lower gastrointestinal bleeding, IIGIB: iatrogenic intraprocedural gastrointestinal bleeding, IDGIB: iatrogenic delayed gastrointestinal bleeding.

A total of 90 out of 1021 patients (8.9%) required admission to the intensive care unit (ICU). The highest rate of ICU admission was observed in the VUGIB group (21.6%), which was significantly higher compared with the NVUGIB, LGIB, IIGIB, and IDGIB groups (Figure 4). The rate of ICU admission in the SBB group (9.1%) was not significantly different from that of the NVUGIB group. ICU admission was uncommon in LGIB (4%) and IDGIB (2%) patients, and no ICU admissions occurred in the IIGIB group. Among patients requiring ICU-level care, the in-hospital mortality rate was 32.2% (29/90).

#### 3.2.4. Length of Hospitalization

**Figure 5:**
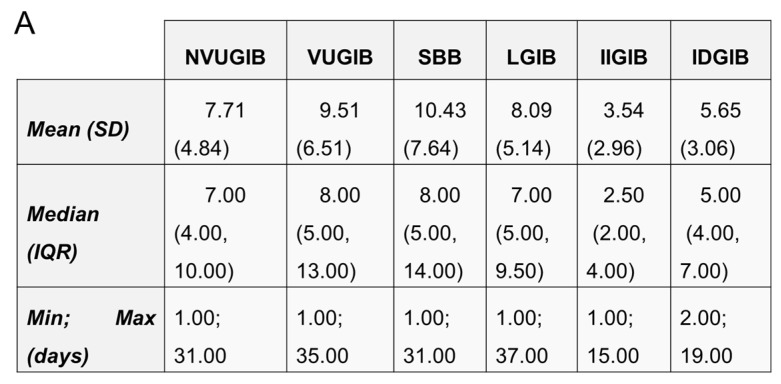

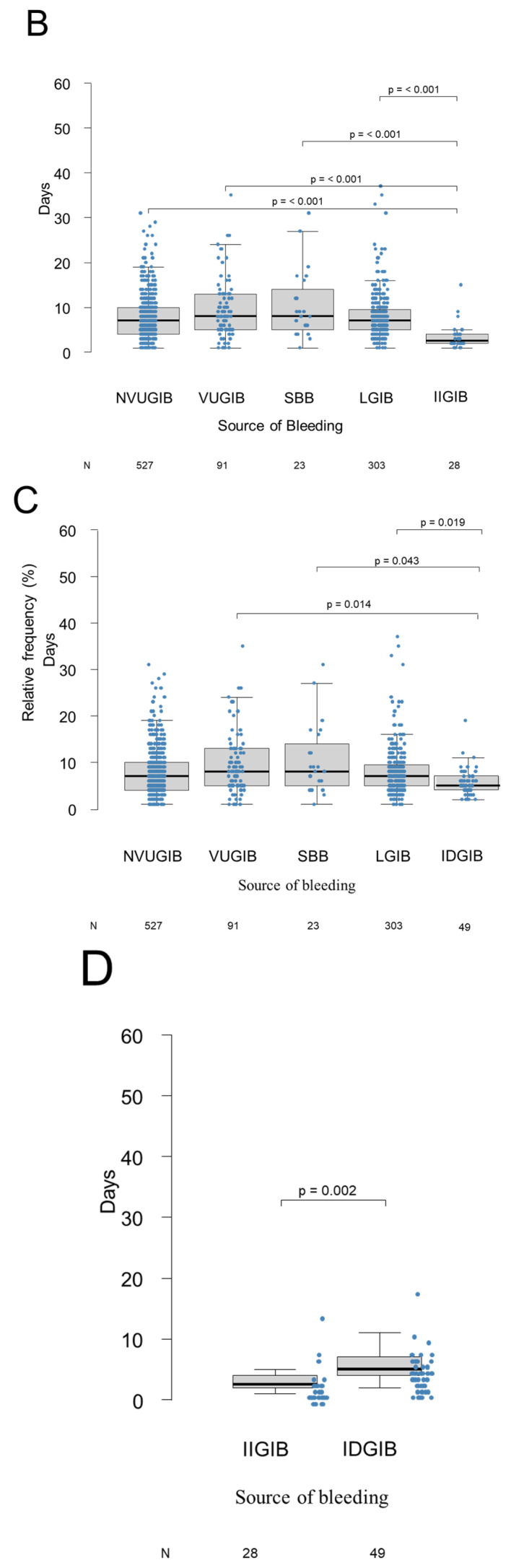
(**A**): Shows the mean, median, minimum, and maximum days of LoH. LoH: length of hospitalization, NVUGIB: non-variceal upper gastrointestinal bleeding, VUGIB: variceal upper gastrointestinal bleeding, SBB: small bowel bleeding, LGIB: lower gastrointestinal bleeding, IIGIB: iatrogenic intraprocedural gastrointestinal bleeding, IDGIB: iatrogenic delayed gastrointestinal bleeding, SD: standard deviation, IQR: interquartile range, Min: minimum, Max: maximum; (**B**): Shows the length of hospitalization and the differences between the acute gastrointestinal bleeding groups. NVUGIB: non-variceal upper gastrointestinal bleeding, VUGIB: variceal upper gastrointestinal bleeding, SBB: small bowel bleeding, LGIB: lower gastrointestinal bleeding, IIGIB: iatrogenic intraprocedural gastrointestinal bleeding, IDGIB: iatrogenic delayed gastrointestinal bleeding; (**C**): Shows the length of hospitalization and the differences between the acute gastrointestinal bleeding groups. NVUGIB: non-variceal upper gastrointestinal bleeding, VUGIB: variceal upper gastrointestinal bleeding, SBB: small bowel bleeding, LGIB: lower gastrointestinal bleeding, IIGIB: iatrogenic intraprocedural gastrointestinal bleeding, IDGIB: iatrogenic delayed gastrointestinal bleeding; (**D**): Shows the length of hospitalization and the differences between the acute gastrointestinal bleeding groups. NVUGIB: non-variceal upper gastrointestinal bleeding, VUGIB: variceal upper gastrointestinal bleeding, SBB: small bowel bleeding, LGIB: lower gastrointestinal bleeding, IIGIB: iatrogenic intraprocedural gastrointestinal bleeding, IDGIB: iatrogenic delayed gastrointestinal bleeding.

The overall median length of hospital stay was 7 days (IQR, 4–10 days). The longest hospitalization duration was observed in the VUGIB and SBB groups, with a median of 8 days (VUGIB: IQR 5–13; SBB: IQR 5–14). Patients in the NVUGIB and LGIB groups had a median hospital stay of 7 days (NVUGIB: IQR 4–10; LGIB: IQR 5–9.5) (Figure 5A).

In contrast, the shortest hospitalization durations were seen in the iatrogenic bleeding groups. The IIGIB group had a median length of stay of 2.5 days (IQR 2–4), which was significantly shorter than in all other bleeding subtypes (Figure 5B). The IDGIB group had a median length of stay of 5 days (IQR 4–7), which was significantly shorter than in the VUGIB, SBB, and LGIB groups (Figure 5C). Furthermore, a significant difference was observed between the two iatrogenic GIB groups, with IIGIB patients having a markedly shorter stay than IDGIB patients (Figure 5D).

#### 3.2.5. Endoscopic Procedures and Interventions

**Figure 6:**
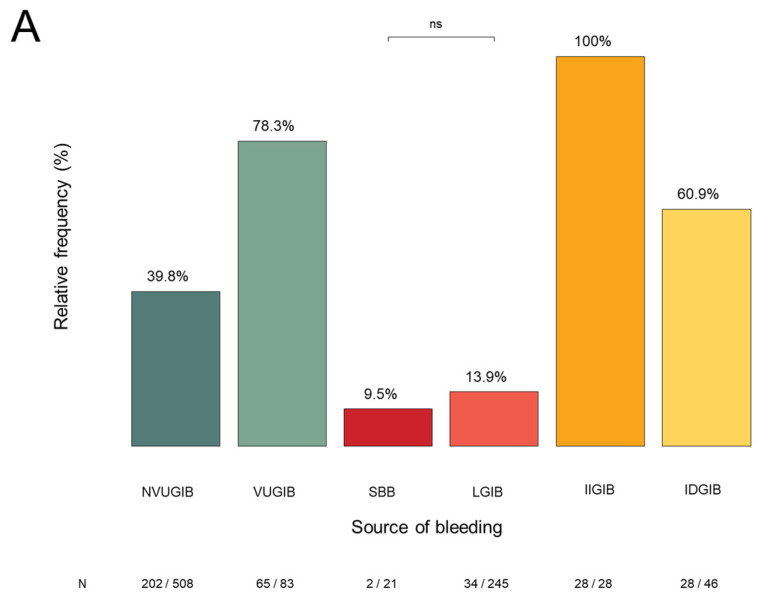

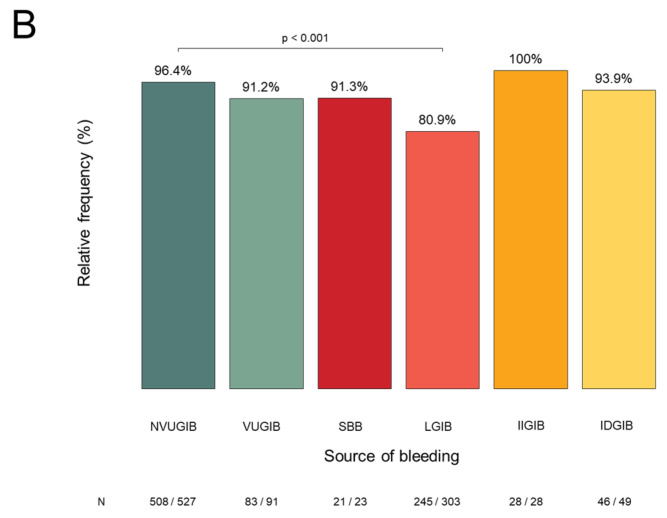
(**A**): The rate of the endoscopic procedures during hospitalization and the significant differences between the bleeding groups. NVUGIB: non-variceal upper gastrointestinal bleeding, VUGIB: variceal upper gastrointestinal bleeding, SBB: small bowel bleeding, LGIB: lower gastrointestinal bleeding, IIGIB: iatrogenic intraprocedural gastrointestinal bleeding, IDGIB: iatrogenic delayed gastrointestinal bleeding; (**B**): The rate of the endoscopic haemostatic interventions during hospitalization and the significant differences between the bleeding groups. NVUGIB: non-variceal upper gastrointestinal bleeding, VUGIB: variceal upper gastrointestinal bleeding, SBB: small bowel bleeding, LGIB: lower gastrointestinal bleeding, IIGIB: iatrogenic intraprocedural gastrointestinal bleeding, IDGIB: iatrogenic delayed gastrointestinal bleeding, ns: not significant.

During hospitalization, 931 of 1021 patients (91%) underwent endoscopic examination. A significant difference was observed in the frequency of endoscopy between the NVUGIB and LGIB groups. In the remaining 9% of patients, endoscopy could not be performed because the patient’s clinical condition did not permit safe intervention, or the patient/next of kin declined the procedure. (Figure 6A) Among the 931 patients who underwent endoscopy, 572 received endoscopic haemostatic intervention. The frequency of endoscopic intervention differed significantly between groups, except for the comparison between the SBB and LGIB groups (Figure 6B).

#### 3.2.6. Need for RBC Transfusion

**Figure 7:**
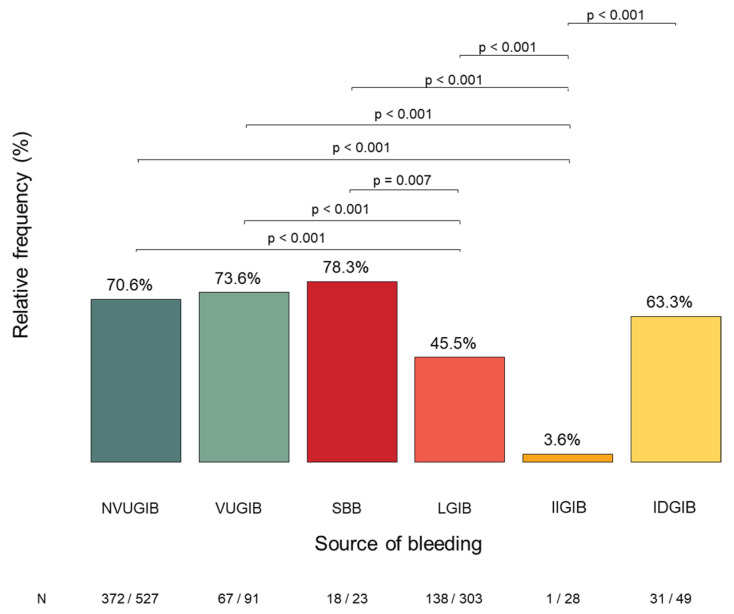
Shows the red blood cell transfusion during hospitalization and the significant differences between the bleeding groups. NVUGIB: non-variceal upper gastrointestinal bleeding. VUGIB: variceal upper gastrointestinal bleeding. SBB: small bowel bleeding. LGIB: lower gastrointestinal bleeding. IIGIB: iatrogenic intraprocedural gastrointestinal bleeding. IDGIB: iatrogenic delayed gastrointestinal bleeding.

RBC transfusion was required in 627 of 1021 patients (61.4%) during hospitalization, with a mean transfused volume of 2.62 units (SD 3.27) and a median of 2.0 units (IQR 0.0–4.0). The frequency of RBC transfusion differed significantly across bleeding subgroups, with the highest rates observed in the SBB and VUGIB groups and the lowest in the IIGIB group. LGIB patients also required RBC transfusion significantly less frequently than patients with NVUGIB, VUGIB, or SBB (Figure 7).

#### 3.2.7. Malignant Lesions as a Source of GIB

**Figure 8:**
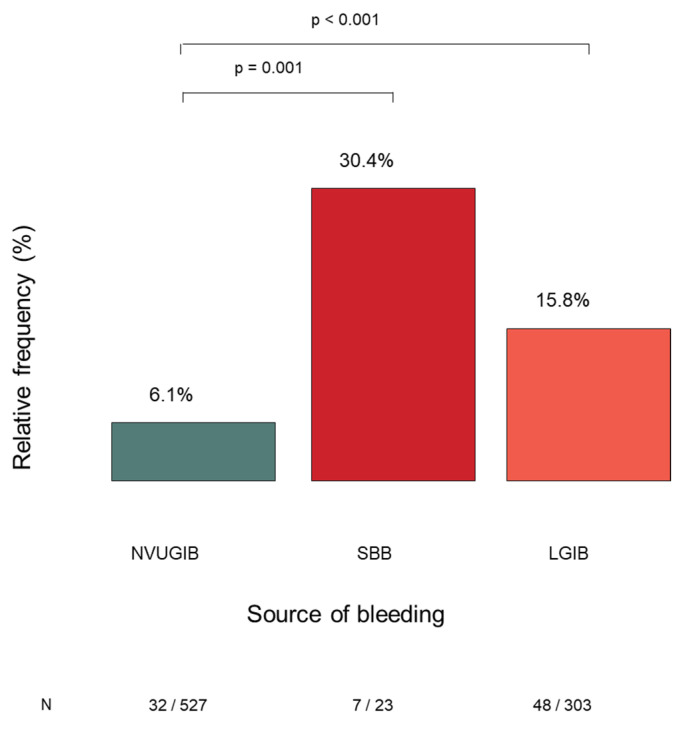
The relative rates of malignant tumors as a source of acute gastrointestinal bleeding and the significant differences between the bleeding groups. NVUGIB: non-variceal upper gastrointestinal bleeding. SBB: small bowel bleeding. LGIB: lower gastrointestinal bleeding.

Malignancy was identified as the source of GIB in 87 of 1021 patients (8.5%). The frequency of malignancy-related bleeding differed substantially across bleeding subtypes. Malignant lesions were detected in 6.1% of NVUGIB cases (32/527), 30.4% of SBB cases (7/23), and 15.8% of LGIB cases (48/303). The proportion of malignancy as the bleeding source was significantly higher in the SBB and LGIB groups compared with the NVUGIB group (Figure 8). No malignancy-related bleeding was identified in the VUGIB, IIGIB, or IDGIB groups.

#### 3.2.8. Need for Surgery

**Figure 9:**
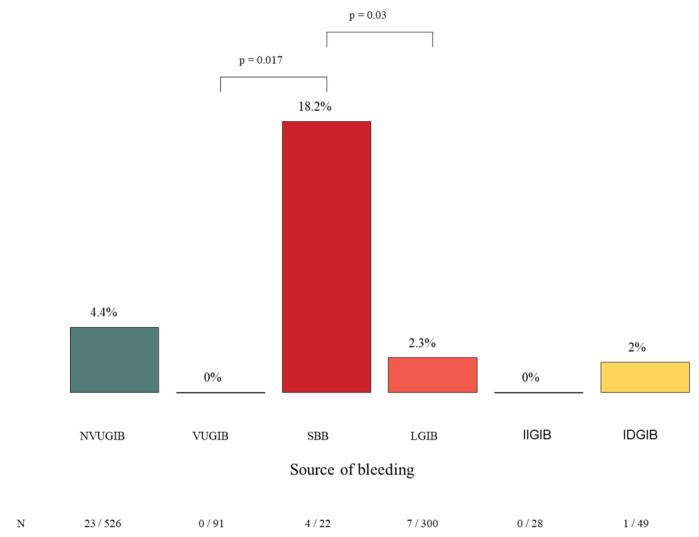
The rate of surgical interventions and the significant differences between the bleeding groups. NVUGIB: nonvariceal upper gastrointestinal bleeding. VUGIB: variceal upper gastrointestinal bleeding. SBB: small bowel bleeding. LGIB: lower gastrointestinal bleeding. IIGIB: iatrogenic intraprocedural gastrointestinal bleeding. IDGIB: iatrogenic delayed gastrointestinal bleeding.

Emergency surgery was required in 35 of 1021 patients (3.4%). The need for surgical intervention varied significantly between bleeding subtypes. The highest rate was observed in the SBB group, where 18.2% (4/22) of patients required surgery, which was significantly higher compared with that in the NVUGIB and LGIB groups (Figure 9). In contrast, surgical intervention was rare in the NVUGIB (4.1%) and LGIB (2.3%) groups. No patients in the VUGIB or IIGIB group required surgery. In the IDGIB group, surgical intervention was necessary in 2% (1/49) of cases.

## 4. Discussion

Across all bleeding subtypes, patients in this cohort were predominantly older adults with a high prevalence of multimorbidity and frequent use of antiplatelet or anticoagulant medication. This constellation of advanced age, polypharmacy, and chronic systemic disease highlights the clinical vulnerability of patients presenting with GIB. Consistent with previous cohort observations, mortality clustered among individuals with significant comorbidity rather than in those experiencing isolated hemorrhage, indicating that physiological reserve and systemic illness are major determinants of outcomes [8,11]. This may explain why several population-based studies have reported only limited mortality reduction despite advances in endoscopic hemostasis, as outcomes depend more on patient frailty than bleeding control alone.

This study provides a comparative analysis of acute gastrointestinal bleeding across six subgroups using unified outcome definitions and consistent evaluation criteria. By examining NVUGIB, VUGIB, SBB, LGIB, and two distinct iatrogenic bleeding groups (IIGIB and IDGIB) within the same analytical framework, our work offers a broader perspective than previous national audits, which typically evaluated only upper or lower bleeding separately.

Patients with variceal upper gastrointestinal bleeding demonstrated the most severe clinical course, with higher rates of hemodynamic instability, transfusion requirement, ICU admission, and in-hospital mortality. These findings are consistent with large observational studies and reflect the effect of portal hypertension and impaired hepatic reserve, where bleeding often precipitates multi-organ decompensation rather than acting as the direct cause of death [8,12,13,14]. In contrast, non-variceal upper and lower gastrointestinal bleeding showed more moderate severity in line with epidemiological reports [5,8,9]. Thus, in variceal bleeding, the hemorrhage primarily functions as a trigger for systemic deterioration, which may explain the higher ICU utilization and early mortality clustering observed in our cohort compared with mixed GIB populations.

Small bowel bleeding represented the least frequent subtype. Diagnostic evaluation is often prolonged, contributing to longer hospitalization and increased healthcare utilization, as described in ACG guidelines [4]. A substantial proportion of cases were associated with neoplastic lesions, supporting the need for systematic evaluation and early capsule or device-assisted enteroscopy [15,16]. The prolonged hospitalization likely reflects diagnostic delay rather than disease severity.

Mortality occurred predominantly early during hospitalization, with more than half of deaths within the first week, consistent with registry data [11]. Only one-third of deaths were directly attributable to bleeding, while most resulted from sepsis, organ failure, malignancy, or decompensation of chronic disease. This indicates that acute GIB frequently represents a precipitating event in a vulnerable population rather than an isolated lethal condition [9,17,18]. Differences from procedure-based cohorts likely arise from the inclusion of consecutive emergency admissions.

Resource utilization closely followed disease severity. Transfusion was most common in VUGIB and SBB, while ICU admission mainly affected severe upper gastrointestinal bleeding.

These findings suggest that early identification of bleeding subtypes may improve monitoring strategies, admission triage, and allocation of hospital resources in clinical practice.

A strength of this study is the standardized data collection with predefined variables and multi-level validation, enabling reliable comparison across bleeding subtypes. To our knowledge, this represents the largest Hungarian cohort analysis of acute gastrointestinal bleeding and provides real-world insight into current management. Separate characterization of intraprocedural and delayed iatrogenic bleeding offers additional clinically relevant information rarely reported previously.

To our knowledge, this study represents one of the few comprehensive consecutive cohort-based comparisons across all major subtypes of gastrointestinal bleeding, applying uniform definitions within a systematically validated dataset. The results demonstrate clinically meaningful differences between bleeding entities in real-world practice, supporting a more subtype-oriented approach to management. Rather than establishing causal relationships, the analysis provides a descriptive reference framework for future prospective and multicenter investigations.

Future studies should integrate bleeding subtype and comorbidity burden into prognostic models, as current scores mainly rely on admission physiology. Prospective multicenter studies including post-discharge outcomes are needed to determine whether subtype-based management improves survival.

This study has limitations. Data derived from two tertiary centres may limit generalizability. The small bowel bleeding subgroup was small, reducing statistical power, and only in-hospital outcomes were available.

## 5. Conclusions

In summary, this descriptive cohort study provides a comprehensive real-world overview of acute GIB with uniform outcome evaluation across six clinically meaningful subtypes. The findings demonstrate clinically relevant differences in early mortality patterns and comorbidity burden between bleeding entities. These descriptive results support a more structured, subtype-oriented approach to clinical assessment and underscore the need for prospective multicentre studies including post-discharge follow-up.

## Figures and Tables

**Table 1:** Presents the absolute numbers and percentages of each variable across the six GIB subgroups, together with the corresponding *p*-values from the statistical comparisons. GIB: acute gastrointestinal bleeding. No: number of patients. NVUGIB: non-variceal upper gastrointestinal bleeding, VUGIB: variceal upper gastrointestinal bleeding, SBB: small bowel bleeding, LGIB: lower gastrointestinal bleeding, IIGIB: iatrogenic intraprocedural gastrointestinal bleeding, IDGIB: iatrogenic delayed gastrointestinal bleeding, SD: standard deviation, IQR: interquartile range. ASA: acetylsalicylic acid. NSAID: Non-Steroidal Anti-Inflammatory Drug, LMWH: low molecular weight heparin, DOAC: direct oral anticoagulant, PPI: proton pump inhibitors. T2DM: type 2 diabetes mellitus, T1DM: type one diabetes mellitus. TIA: transient ischaemic attack. COPD: chronic obstructive lung disease. N.A.: not applicable. Bold text indicates the main variable headings within the table.

GIB Subtypes	NVUGIB	VUGIB	SBB	LGIB	IIGIB	IDGIB	*p*-Values
No, %	527/1021; 51.03%	91/1021; 8.9%	23/1021; 2.25%	303/1021; 29.68%	28/1021; 2.74%	49/1021; 4.8%	N.A.
**Gender (No, %)**							<0.001
Male	327 (62%)	71 (78%)	13 (57%)	152 (50%)	19 (68%)	29 (59%)	
Female	200 (38%)	20 (22%)	10 (43%)	151 (50%)	9 (32%)	20 (41%)	
**Age (years)**							<0.001
Mean (SD)	69.96 (12.87)	58.93 (9.74)	73.00 (14.53)	72.18 (14.15)	68.39 (11.90)	65.59 (13.98)	
Median (IQR)	70.00 (61.00, 80.00)	59.00 (54.00, 65.00)	78.00 (67.00, 82.50)	75.00 (65.00, 83.00)	66.50 (62.75, 76.75)	69.00 (58.00, 76.00)	
**Age group distribution (No, %)**							<0.0001
18–44	15 (2.8%)	5 (5.5%)	1 (4.3%)	21 (6.9%)	1 (3.6%)	4 (8.2%)	
45–64	165 (31%)	62 (68%)	3 (13%)	54 (18%)	7 (25%)	14 (29%)	
65–79	201(38%)	23 (25%)	11 (48%)	121 (40%)	14 (50%)	25 (51%)	
≥80	146 (28%)	1 (1.1%)	8 (35%)	107 (35%)	6 (21%)	6 (12%)	
**Patient previously in GIB registry (No, %)**	30 (5.7%)	18 (20%)	4 (17%)	23 (7.6%)	1 (3.6%)	9 (18%)	<0.001
**Where was the bleeding detected (No, %)**							<0.001
Community-onset	437 (83%)	79 (87%)	18 (78%)	256 (84%)	-	33 (67%)	
Inpatient	90 (17%)	12 (13%)	5 (22%)	47 (16%)	-	16 (33%)	
**Risk factors (No, %)**							
Recent alcohol consumption	200 (38%)	49 (54%)	10 (43%)	93 (31%)	13 (46%)	15 (31%)	0.057
Past alcohol consumption	67 (13%)	28 (31%)	1 (4.3%)	26 (8.6%)	2 (7.1%)	6 (12%)	0.018
Recent smoking	122 (23%)	25 (27%)	2 (8.7%)	40 (13%)	4 (14%)	5 (10%)	0.001
Past smoking	112 (21%)	23 (25%)	4 (17%)	58 (19%)	8 (29%)	8 (16%)	0.076
**Medications on admission (No, %)**							
Vitamin K antagonists	64 (12%)	2 (2.2%)	3 (13%)	32 (11%)	0 (0%)	4 (8.2%)	0.034
LMWH	36 (6.8%)	7 (7.7%)	0 (0%)	37(12%)	0	11 (22%)	<0.001
DOACs	52 (9.9%)	1 (1.1%)	3 (13%)	66 (22%)	3 (11%)	8 (16%)	<0.001
ASA	100 (19%)	6 (6.6%)	4 (17%)	81 (27%)	8 (29%)	8 (16%)	<0.001
Non-ASA antiplatelet agents	60 (11%)	3 (3.3%)	7 (30%)	47 (16%)	5 (18%)	5 (10%)	<0.001
NSAIDs	102 (19%)	5 (5.5%)	3 (13%)	34 (11%)	0	4 (8.2%)	<0.001
Corticosteroids	18 (3.4%)	3 (3.3%)	1 (4.3%)	15 (5.0%)	3 (11%)	1 (2.0%)	0.371
Gastroprotective medications (PPIs, H2-receptor antagonists, etc.)	209 (40%)	59 (65%)	16 (70%)	180 (59%)	13 (46%)	34 (69%)	<0.001
**Previous manifest GIB in history (No, %)**	136 (26%)	52 (57%)	10 (43%)	102 (34%)	7 (25%)	21 (43%)	<0.001
**Hemodynamic instability on admission (No, %)**	111 (21%)	16 (18%)	2 (8.7%)	22 (7.3%)	2 (7.1%)	7 (14%)	<0.001
**Co-morbidities (No, %)**							
Hypertension	368 (70%)	50 (55%)	19 (83%)	235 (78%)	19 (68%)	35 (71%)	0.001
Acute myocardial infarction	27 (5.1%)	4 (4.4%)	2 (8.7%)	23 (7.6%)	2 (7.1%)	3 (6.1%)	0.747
Atrial fibrillation or flutter	116 (22%)	6 (6.6%)	5 (22%)	90 (30%)	4 (14%)	15 (31%)	<0.001
Permanent pacemaker implanted	20 (3.8%)	0 (0%)	2 (8.7%)	20 (6.6%)	2 (7.1%)	4 (8.2%)	0.017
Ischemic heart disease	301 (57%)	56 (62%)	11 (48%)	162 (53%)	14 (50%)	31 (63%)	0.009
Chronic heart failure	72 (14%)	4 (4.4%)	5 (22%)	48 (16%)	1 (3.6%)	7 (14%)	0.021
T2DM	128 (24%)	34 (37%)	9 (39%)	91 (30%)	14 (50%)	13 (27%)	0.999
T1DM	2 (0.4%)	0	0	1 (0.3%)	0	0	0.999
Stroke	58 (11%)	1 (1.1%)	0 (0%)	63 (21%)	2 (7.1%)	4 (8.2%)	<0.001
TIA	14 (2.7%)	0 (0%)	2 (8.7%)	18 (5.9%)	0 (0%)	0 (0%)	0.012
Chronic renal failure	170 (32%)	14 (15%)	7 (30%)	122 (40%)	9 (32%)	14 (29%)	<0.001
Hemodialysis	8 (1.5%)	0 (0%)	0 (0%)	9 (3.0%)	0 (0%)	0 (0%)	0.819
Pulmonary diseases	105 (20%)	12 (13%)	10 (43%)	58 (19%)	3 (11%)	10 (20%)	0.045
COPD	42 (8.0%)	3 (3.3%)	5 (22%)	28 (9.2%)	3 (11%)	4 (8.2%)	0.100
Pulmonary embolism	27 (5.1%)	1 (1.1%)	1 (4.3%)	31 (10%)	1 (3.6%)	2 (4.1%)	0.014
Deep vein thrombosis	31 (5.9%)	2 (2.2%)	0 (0%)	27 (8.9%)	2 (7.1%)	4 (8.2%)	0.194
Peripheral Arterial Disease	33 (6.3%)	1 (1.1%)	1 (4.3%)	14 (4.6%)	1 (3.6%)	3 (6.1%)	0.375
Liver disease	100 (19%)	72 (79%)	4 (17%)	29 (9.6%)	2 (7.1%)	10 (20%)	<0.001
Cirrhosis	61 (12%)	69 (76%)	0 (0%)	11 (3.6%)	0 (0%)	7 (14%)	<0.001
Malignant tumour in the medical history	134 (25%)	16 (18%)	12 (52%)	84 (28%)	10 (36%)	13 (27%)	0.021
Peptic ulcer disease in the medical history	76 (14%)	16 (18%)	3 (13%)	21 (6.9%)	2 (7.1%)	1 (2.0%)	<0.001

**Table 2:** The in-hospital outcomes are shown as absolute numbers and percentages across the six gastrointestinal bleeding subgroups. NVUGIB: non-variceal upper gastrointestinal bleeding. VUGIB: variceal upper gastrointestinal bleeding. SBB: small bowel bleeding. LGIB: lower gastrointestinal bleeding. IIGIB: iatrogenic intraprocedural gastrointestinal bleeding. IDGIB: iatrogenic delayed gastrointestinal bleeding. ICU: intensive care unit. LoH: length of hospitalization. SD: standard deviation. IQR: interquartile range. RBC: red blood cell. Bold text indicates the main variable headings within the table.

	NVUGIB	VUGIB	SBB	LGIB	IIGIB	IDGIB
**In-hospital mortality (No, %)**	65 (12.3%)	20 (22%)	1 (4.3%)	18 (5.9%)	0	4 (8.2%)
**In-hospital rebleeding (No, %)**	32 (6.1%)	9 (9.9%)	0	9 (3.0%)	0	4 (8.2%)
**Need for ICU (No, %)**	56 (11%)	19 (21%)	2 (8.7%)	12 (4.0%)	0	1 (2.0%)
**LoH**						
Mean (days, SD)	7.71 (4.84)	9.51 (6.51)	10.43 (7.64)	8.09 (5.14)	3.54 (2.96)	5.65 (3.06)
Median (days, IQR)	7.00 (4.00, 10.00)	8.00 (5.00, 13.00)	8.00 (5.00, 14.00)	7.00 (5.00, 9.50)	2.50 (2.00, 4.00)	5.00 (4.00, 7.00)
Minimum; Maximum (days)	1.00; 31.00	1.00; 35.00	1.00; 31.00	1.00; 37.00	1.00; 15.00	2.00; 19.00
**Endoscopy during hospitalization (No, %)**	508 (96%)	83 (91%)	21 (91%)	245 (81%)	-	46 (94%)
**Endoscopic intervention (No, %)**	202 (38%)	65 (71%)	2 (8.7%)	34 (11%)	28 (100%)	28 (57%)
**RBC transfusion (No, %)**	372 (71%)	67 (74%)	18 (78%)	138 (46%)	1 (3.6%)	31 (63%)
**Malignant lesion as source of bleeding (No, %)**	32 (6.1%)	0	7 (30%)	48 (16%)	0	0
**Need for surgery (No, %)**	23 (4.4%)	0	4 (17%)	7 (2.3%)	0	1 (2.0%)

## Data Availability

Detailed data on patient characteristics, comorbidities, medication, treatments, procedures, and outcomes were pro- and retrospectively gathered in an online database (https://tm-centre.org/en/research/registries/gib-registry (accessed on 1 March 2026)). The datasets generated and analysed during the current study are not publicly available in the Hungarian Gastrointestinal Bleeding Registry but are available from the corresponding author on reasonable request.

## Supplementary Materials

The following supporting information can be downloaded at: https://www.mdpi.com/article/10.3390/jcm15051998/s1, Table S1: Strengthening the Reporting of Observational Studies in Epidemiology (STROBE) 2007 checklist; Table S2: Definitions used in this study; Table S3: Data quality and completeness of variables included in Table 1 and Table 2.

## Abbreviations

The following abbreviations are used in this manuscript:

ACG	American College of Gastroenterology
ASA	Acetylsalicylic acid
COPD	Chronic obstructive pulmonary disease
CT	Computed tomography
DOAC	Direct oral anticoagulant
ESGE	European Society of Gastrointestinal Endoscopy
GI	Gastrointestinal
GIB	Gastrointestinal bleeding
ICU	Intensive care unit
IDGIB	Iatrogenic delayed gastrointestinal bleeding
IIGIB	Iatrogenic intraprocedural gastrointestinal bleeding
IQR	Interquartile range
LGIB	Lower gastrointestinal bleeding
LMWH	Low molecular weight heparin
LoH	Length of hospitalization
MR	Magnetic resonance imaging
NSAID	Non-steroidal anti-inflammatory drug
NVUGIB	Non-variceal upper gastrointestinal bleeding
PPI	Proton pump inhibitor
RBC	Red blood cell
SBB	Small bowel bleeding
SD	Standard deviation
STROBE	Strengthening the Reporting of Observational Studies in Epidemiology
TIA	Transient ischaemic attack
T1DM	Type 1 diabetes mellitus
T2DM	Type 2 diabetes mellitus
VUGIB	Variceal upper gastrointestinal bleeding
