# Supplementary material for: Distinct Clinical and Outcome Profiles Across Six Subtypes of Acute Gastrointestinal Bleeding: A Comprehensive Analysis of 1021 Patients
Source: J Clin Med. 2026 Mar 5;15(5):1998. doi: 10.3390/jcm15051998 (PMC12985898; doi:10.3390/jcm15051998)
Supplement: Supplementary file 1 [file jcm-15-01998-s001.zip › jcm-4129792-supplementary.pdf]

SUPPLEMENTARY MATERIAL

Supplementary Table 1. Strengthening the Reporting of Observational Studies in Epidemiology (STROBE) 2007 checklist

	Item No	Recommendation	Relevant text from manuscript
**Title and abstract**	1	(*a*) Indicate the study's design with a commonly used term in the title or the abstract	cohort analysis
		(*b*) Provide in the abstract an informative and balanced summary of what was done and what was found	The abstract provides a concise and balanced summary of an ambidirectional, multicentre registry-based cohort study of 1,021 consecutive cases of gastrointestinal bleeding. It specifies the study design, population, and main objectives, and summarizes the principal clinical outcomes including in-hospital mortality, in-hospital rebleeding, ICU admission, length of hospitalization, endoscopic and surgical interventions, and transfusion requirements. The results are presented clearly, highlighting significant differences across bleeding subtypes, while the conclusions emphasize the heterogeneity of outcomes and clinical characteristics among these groups.
**Introduction**			
Background/rationale	2	Explain the scientific background and rationale for the investigation being reported	The introduction clearly explains that acute gastrointestinal bleeding (GIB) represents a major medical emergency with substantial morbidity and mortality, yet most previous studies have focused on specific subtypes (e.g., upper, lower, or variceal bleeding) rather than analysing all categories within a single framework. The rationale for this study was to address this knowledge gap by providing a comprehensive, real-world overview of patient characteristics, management strategies, and outcomes across six major GIB subtypes, based on data from a multicentre registry.
Objectives	3	State specific objectives, including any prespecified hypotheses	The primary objective was to provide a comprehensive comparison of patient characteristics, management patterns, and clinical outcomes across six major gastrointestinal bleeding (GIB) subtypes: non-variceal upper (NVUGIB), variceal upper (VUGIB), small bowel (SBB), lower (LGIB), iatrogenic intraprocedural (IIGIB), and iatrogenic delayed (IDGIB) bleeding. Specific aims included describing demographics, comorbidities, medication use, endoscopic management, and key outcomes (in-hospital mortality, rebleeding, ICU admission, and length of hospitalization). The prespecified hypothesis was that different GIB subtypes represent distinct clinical entities with substantial variation in presentation, management, and outcomes.
**Methods**			
Study design	4	Present key elements of study design early in the paper	The key elements of the study design are clearly presented both in the abstract and at the beginning of the Methods section. The study is described as an ambidirectional, multicentre, registry-based cohort analysis including 1,021 consecutive cases of acute gastrointestinal bleeding (GIB) collected between October 2019 and April 2022 across six Hungarian tertiary centres. Patients were classified into six predefined GIB subtypes, and outcomes were compared across groups.
Setting	5	Describe the setting, locations, and relevant dates, including periods of recruitment, exposure, follow-up, and data collection	The study was conducted in two tertiary referral hospitals in Hungary (Pécs University Hospital and Székesfehérvár Hospital). Consecutive patients hospitalized with acute gastrointestinal bleeding were enrolled between 6 October 2019 and 7 April 2022. Data were collected using a standardized electronic case report form, with both prospective and retrospective entries depending on the site. The observation period covered the full duration of the index hospitalization, during which detailed clinical outcomes were recorded, including in-hospital mortality, in-hospital rebleeding, intensive care unit (ICU) admission, length of hospitalization (LoH), performance of endoscopy, use and type of endoscopic haemostatic intervention, need for surgical intervention, identification of malignant lesions as the source of bleeding, and red blood cell (RBC) transfusion.
Participants	6	(*a*) Give the eligibility criteria, and the sources and methods of selection of participants.	All adult patients (≥18 years) hospitalized with acute gastrointestinal bleeding were eligible for inclusion, whether bleeding was community-onset or developed during hospitalization. Consecutive cases were identified from two tertiary referral hospitals (Pécs University Hospital and Székesfehérvár Hospital) and entered into an institutional GIB registry. Data collection followed an ambidirectional approach, combining

		Describe methods of follow-up	prospective recording of ongoing cases and retrospective inclusion of earlier admissions using a uniform electronic case report form and standardized diagnostic criteria.<br><br>The follow-up period covered the entire duration of the index hospitalization, during which daily data entry was performed for each patient to document clinical status, management, and hospital course in real time.
		(*b*) For matched studies, give matching criteria and number of exposed and unexposed	N.A.
Variables	7	Clearly define all outcomes, exposures, predictors, potential confounders, and effect modifiers. Give diagnostic criteria, if applicable	All outcomes, exposures, and predictors were predefined and are described in detail in Supplementary Table 2.
Data sources/ measurement	8*	For each variable of interest, give sources of data and details of methods of assessment (measurement). Describe comparability of assessment methods if there is more than one group	Detailed data on patient characteristics, comorbidities,<br><br>medication, treatments, procedures, and outcomes were pro- and<br><br>retrospectively gathered in an online database (https://tmcentre.org/en/research/registries/gib-registry).
Bias	9	Describe any efforts to address potential sources of bias	N.A.
Study size	10	Explain how the study size was arrived at	The study size was determined by the number of consecutive cases of acute gastrointestinal bleeding recorded in the registry during the predefined inclusion period (6 October 2019 to 7 April 2022). No formal sample size calculation was performed, as all eligible patients hospitalized with GIB at the two participating tertiary centers were included.
Quantitative variables	11	Explain how quantitative variables were handled in the analyses. If applicable, describe which groupings were chosen and why	Quantitative variables were analyzed as continuous data and are presented as mean ± standard deviation (SD) or median with interquartile range (IQR), depending on distribution.<br><br>For group comparisons, one-way ANOVA was applied to continuous variables with normally distributed data, and post hoc pairwise differences were assessed using Tukey's test.<br><br>Continuous variables with skewed distributions were summarized using medians and IQRs and compared using non-parametric methods where appropriate.<br><br>Age was additionally grouped into predefined clinical categories (18–44, 45–64, 65–79, and ≥80 years) to facilitate descriptive interpretation across gastrointestinal bleeding subtypes.
Statistical methods	12	(*a*) Describe all statistical methods, including those used to control for confounding	Descriptive statistics were applied to summarize the data. Continuous variables are presented as mean ± standard deviation (SD) or median and interquartile range (IQR), as appropriate, while categorical variables are reported as counts and percentages.<br><br>Group comparisons for continuous variables were performed using one-way ANOVA; where significant, pairwise post hoc comparisons were conducted using Tukey's test. Categorical variables were compared using Pearson's chi-squared test or Fisher's exact test when expected cell counts were low; pairwise comparisons were adjusted using the Benjamini–Hochberg procedure to control the false discovery rate. Survival analysis for in-hospital mortality was conducted using Kaplan–Meier estimates, with the length of hospital stay serving as the time variable. Differences between groups were assessed using the log-rank test. No multivariable adjustment was performed, as the primary aim

			was descriptive comparison among predefined bleeding subtypes. However, uniform definitions, standardized data collection, and consistent statistical procedures were applied across both centers to minimize confounding. A two-tailed p-value < 0.05 was considered statistically significant. All analyses were performed using R (version 4.4.2; R Foundation for Statistical Computing, Vienna, Austria).
		(*b*) Describe any methods used to examine subgroups and interactions	Subgroup analyses were predefined according to gastrointestinal bleeding subtype (NVUGIB, VUGIB, SBB, LGIB, IIGIB, and IDGIB). Comparative analyses of baseline characteristics and clinical outcomes were performed across these six subgroups using the same statistical procedures described above. Pairwise post hoc tests (Tukey's and Benjamini–Hochberg correction) were applied to identify significant differences between specific subgroups. No formal interaction analyses or multivariable modelling were performed, as the study was primarily descriptive and exploratory in nature.
		(*c*) Explain how missing data were addressed	N.A
		(*d*) If applicable, explain how loss to follow-up was addressed	N.A
		(*e*) Describe any sensitivity analyses	N.A
**Results**			
Participants	13*	(a) Report numbers of individuals at each stage of study—eg numbers potentially eligible, examined for eligibility, confirmed eligible, included in the study, completing follow-up, and analysed	Between 6 October 2019 and 7 April 2022, a total of 1,021 consecutive patients hospitalized with acute gastrointestinal bleeding were enrolled from two tertiary referral centres. All eligible patients during the inclusion period were entered into the registry and included in the present analysis. No exclusions were applied, and complete follow-up data were available for all participants throughout the index hospitalization.
		(b) Give reasons for non-participation at each stage	There were no cases of non-participation or loss to follow-up.<br><br>All patients hospitalized with acute gastrointestinal bleeding during the study period were included in the registry and followed throughout the entire hospital stay.
		(c) Consider use of a flow diagram	N.A
Descriptive data	14*	(a) Give characteristics of study participants (eg demographic, clinical, social) and information on exposures and potential confounders	Table 1, See each outcome detailed in the manuscript
		(b) Indicate number of participants with missing data for each variable of interest	There were no missing data for any of the variables included in the analysis.<br><br>Data completeness was 100% for all baseline characteristics and outcome measures, as verified in the data quality assessment (Supplementary Table 3).
		(c) Summarise follow-up time (eg, average and total amount)	The follow-up period corresponded to the duration of the index hospitalization for each patient. All 1,021 patients followed until discharge or in-hospital death.
Outcome data	15*	Report numbers of outcome events or	During the study period, a total of 1,021 patients with acute gastrointestinal bleeding were analysed. Overall in-hospital mortality was 10.6% (108/1,021), with the highest

		summary measures over time	rate observed in variceal upper GIB (22%). Rebleeding occurred in 5.3% (54/1,021) of patients, most frequently in VUGIB (9.9%). ICU admission was required in 8.9% (91/1,021), again most commonly among patients with variceal bleeding (21.6%).<br><br>The median length of hospitalization was 7 days (IQR 4–10), which was significantly shorter in iatrogenic GIB compared to other subtypes ($p < 0.05$). Endoscopy was performed in 91% of cases, and endoscopic haemostatic intervention was carried out in 35%. Red blood cell transfusion was administered in 46% of patients. Surgical intervention was required in 3%, while a malignant lesion was identified as the bleeding source in 7%.
Main results	16	(*a*) Give unadjusted estimates and, if applicable, confounder-adjusted estimates and their precision (eg, 95% confidence interval). Make clear which confounders were adjusted for and why they were included	See each outcome detailed in the manuscript
		(*b*) Report category boundaries when continuous variables were categorized	See group formation for the descriptive analysis in the Methods section
		(*c*) If relevant, consider translating estimates of relative risk into absolute risk for a meaningful time period	N.A.
Other analyses	17	Report other analyses done—eg analyses of subgroups and interactions, and sensitivity analyses	Fig. 1-9<br><br>See each outcome detailed in the manuscript
**Discussion**			
Key results	18	Summarise key results with reference to study objectives	The study summarized and compared the clinical characteristics and in-hospital outcomes of 1,021 patients with different subtypes of gastrointestinal bleeding.<br><br>Variceal bleeding was associated with the highest mortality, rebleeding, and ICU admission rates, while iatrogenic bleeding showed the most favorable short-term outcomes and the shortest hospitalization.
Limitations	19	Discuss limitations of the study, taking into account sources of potential bias or imprecision. Discuss both direction and magnitude of any potential bias	This study has several limitations inherent to its ambidirectional, registry-based design.<br><br>Although data collection was standardized and included all consecutive cases, part of the dataset was retrospectively entered, which may introduce minor information bias.<br><br>The analysis was limited to two tertiary centers, which may affect generalizability to other healthcare settings.<br><br>Furthermore, follow-up was restricted to the index hospitalization, so post-discharge outcomes could not be assessed.
Interpretation	20	Give a cautious overall interpretation of results considering objectives, limitations, multiplicity of analyses, results	See detailed in interpretation.

		from similar studies, and other relevant evidence	
Generalisability	21	Discuss the generalisability (external validity) of the study results	See detailed in interpretation
**Other information**			
Funding	22	Give the source of funding and the role of the funders for the present study and, if applicable, for the original study on which the present article is based	Open access funding provided by the University of Pécs. Funding was provided by Tandem Funding of the University of Pécs (granted to Dr Hágendorn, KA-2021-10) and ÚNKP-22-3 New National Excellence Program of the Ministry for Innovation and Technology from the source of the National Research, Development and Innovation Fund (to BT—ÚNKP-23-3-II-PTE-1996).

Supplementary Table 2. Definitions used in this study

Variable name in Table 1	Definition / operational criteria (specific to registry dataset)
Gender (male/female)	Biological sex as documented in the hospital record at admission; percentages represent the sex distribution within each GIB subtype.
Age (years)	Age at the time of hospital admission. Reported as mean (standard deviation) and median (interquartile range).
Age group distribution	Categorized age intervals: 18–44, 45–64, 65–79, and ≥80 years. Percentages indicate the proportion within each subtype.
Patient previously in GIB registry	Patient with at least one prior documented GIB episode already recorded in the registry database.
Where was the bleeding detected	Location of initial bleeding onset: community-onset = symptoms began before hospital admission; inpatient = bleeding occurred ≥24 hours after hospitalization for another diagnosis.
Recent alcohol consumption	Active alcohol consumption within 30 days before hospital admission.
Past alcohol consumption	History of regular alcohol intake discontinued more than 30 days before admission.
Recent smoking	Current active smoking within 30 days prior to admission.
Past smoking	Previous smoking habit discontinued more than 30 days before admission.
Vitamin K antagonists	Regular use of warfarin or acenocoumarol within 7 days prior to admission.
LMWH	Use of low-molecular-weight heparin (therapeutic or prophylactic dose) within 7 days before admission.
DOACs	Use of direct oral anticoagulants (dabigatran, apixaban, rivaroxaban, or edoxaban) within 7 days prior to admission.
ASA	Use of acetylsalicylic acid (any dose or frequency) within 7 days prior to admission.
Non-ASA antiplatelet agents	Use of P2Y12 inhibitors such as clopidogrel, ticagrelor, or prasugrel within 7 days prior to admission.
NSAIDs	Use of non-steroidal anti-inflammatory drugs within 7 days prior to admission.
Corticosteroids	Systemic corticosteroid therapy (oral or parenteral) ongoing at the time of hospital admission.
Gastroprotective medications	Use of proton pump inhibitors or H_2-receptor antagonists within 7 days prior to hospital admission.
Previous manifest GIB in history	Documented prior GIB event confirmed by endoscopy or medical record review.
Hemodynamic instability in admission	Defined as systolic BP <90 mmHg, heart rate >100 bpm, or need for vasopressor support upon arrival.
Hypertension	History of hypertension or current use of antihypertensive medication.
Acute myocardial infarction	History of acute myocardial infarction documented in the medical records.
Atrial fibrillation or flutter	Documented history of atrial fibrillation or atrial flutter.
Permanent pacemaker implanted	Presence of an implanted permanent cardiac pacemaker device prior to admission.
Ischemic heart disease	Documented history of ischemic heart disease

Chronic heart failure	Diagnosis of heart failure with reduced or preserved ejection fraction, supported by echocardiography or clinical criteria.
T2DM	History of type 2 diabetes mellitus documented in the medical records.
T1DM	History of type 1 diabetes mellitus documented in the medical records.
Stroke	Previous cerebrovascular accident confirmed radiologically with residual neurological deficit.
TIA	Transient focal neurological episode lasting <24 hours, without radiological infarction.
Chronic renal failure	eGFR <60 mL/min/1.73m^2 for >3 months or ongoing dialysis treatment.
Hemodialysis	Regular intermittent or continuous dialysis therapy prior to admission.
Pulmonary diseases	Chronic respiratory disorders including COPD, asthma, or other pulmonary disease.
COPD	Clinically diagnosed chronic obstructive pulmonary disease confirmed by spirometry or medical record.
Pulmonary embolism	Objectively confirmed pulmonary embolism on imaging (CT pulmonary angiography or perfusion scan).
Deep vein thrombosis	Confirmed venous thrombosis verified by Doppler ultrasound or CT venography.
Peripheral Arterial Disease	Clinically or radiologically confirmed peripheral atherosclerotic arterial disease.
Liver disease	Chronic hepatic disorder of any etiology (alcoholic, viral, metabolic, or autoimmune).
Cirrhosis	Established cirrhosis confirmed by imaging, laboratory, or clinical features (e.g., ascites, varices).
Malignant tumour in the medical history	History of solid or hematologic malignancy previously diagnosed and recorded in the patient's chart.
Peptic ulcer disease in the medical history	Previous diagnosis of gastric or duodenal ulcer verified by endoscopy or imaging.

GIB: acute gastrointestinal bleeding.

Variable name in Table 2	Definition / operational criteria
In-hospital mortality (No, %)	Death from any cause occurring during the index hospitalisation. The event was recorded irrespective of the underlying cause, provided it occurred before discharge.
In-hospital rebleeding (No, %)	Recurrent overt gastrointestinal bleeding (haematemesis, melaena or haematochezia) after initial haemostasis, confirmed clinically or endoscopically during the same hospital stay.
Need for ICU (No, %)	Admission to an intensive care unit.
Length of hospitalisation (LoH)	Duration from the date of admission to the date of discharge or in-hospital death, expressed in days.
Mean (days, SD)	Arithmetic mean and standard deviation of hospital stay length, expressed in days.
Median (days, IQR)	Median and interquartile range (25th–75th percentile) of hospital stay duration, expressed in days.
Minimum; Maximum (days)	Shortest and longest observed hospital stays within each bleeding subtype.
Endoscopy during hospitalisation (No, %)	Proportion of patients who underwent at least one diagnostic or therapeutic endoscopic examination during the index admission.
Endoscopic intervention (No, %)	Patients who received an active endoscopic haemostatic therapy.
RBC transfusion (No, %)	Patients who received transfusion of packed red blood cells at any time during hospitalisation.
Malignant lesion as source of bleeding (No, %)	Endoscopically and histologically verified malignant lesion identified as the source of bleeding.
Need for surgery (No, %)	Any surgical procedure performed to control or definitively treat the gastrointestinal bleeding during the same hospital stay.

Supplementary Table 3. Data quality and completeness of variables included in Tables 1–2

Variable name	*Category*	*Data completeness (%)*
GIB subtype	Baseline / Classification	100
Age (mean, median, age group)	Demographic	100
Gender	Demographic	100
Location of bleeding onset (community vs in-hospital)	Demographic	100
Recent alcohol consumption	Risk factor	100
Past alcohol consumption	Risk factor	100
Recent smoking	Risk factor	100

Past smoking	Risk factor	100
Vitamin K antagonists	Medication	100
LMWH	Medication	100
DOACs	Medication	100
ASA	Medication	100
Non-ASA antiplatelet agents	Medication	100
NSAIDs	Medication	100
Corticosteroids	Medication	100
Gastroprotective medications	Medication	100
Previous manifest GIB	Clinical history	100
Previous inclusion in GIB registry	Clinical history	100
Haemodynamic instability on admission	Clinical presentation	100
Hypertension	Comorbidity	100
Acute myocardial infarction	Comorbidity	100
Atrial fibrillation / flutter	Comorbidity	100
Permanent pacemaker implanted	Comorbidity	100
Ischaemic heart disease	Comorbidity	100
Chronic heart failure	Comorbidity	100
Type 2 diabetes mellitus	Comorbidity	100
Type 1 diabetes mellitus	Comorbidity	100
Stroke	Comorbidity	100
Transient ischaemic attack (TIA)	Comorbidity	100
Chronic renal failure	Comorbidity	100
Haemodialysis	Comorbidity	100
Pulmonary diseases	Comorbidity	100
COPD	Comorbidity	100
Pulmonary embolism	Comorbidity	100
Deep vein thrombosis	Comorbidity	100
Peripheral arterial disease	Comorbidity	100
Liver disease	Comorbidity	100
Cirrhosis	Comorbidity	100
Malignant tumour in medical history	Comorbidity	100
Peptic ulcer disease in medical history	Comorbidity	100
In-hospital mortality	Outcome	100
In-hospital rebleeding	Outcome	100
ICU admission	Outcome	100
Length of hospitalisation (LoH)	Outcome	100
Endoscopy during hospitalisation	Outcome	100
Endoscopic intervention	Outcome	100
RBC transfusion	Outcome	100
Malignant lesion as source of bleeding	Outcome	100
Need for surgery	Outcome	100
